# Supplementary material for: Water Quality Assessment Bias Associated with Long-Screened Wells Screened across Aquifers with High Nitrate and Arsenic Concentrations
Source: Int J Environ Res Public Health. 2022 Aug 11;19(16):9907. doi: 10.3390/ijerph19169907 (PMC9408386; doi:10.3390/ijerph19169907)
Supplement: Supplementary file 1 [file ijerph-19-09907-s001.zip › ijerph-1805125-supplementary.pdf]

Content:

Figure: S1-S6
Tables: S1-S8
Text: S1

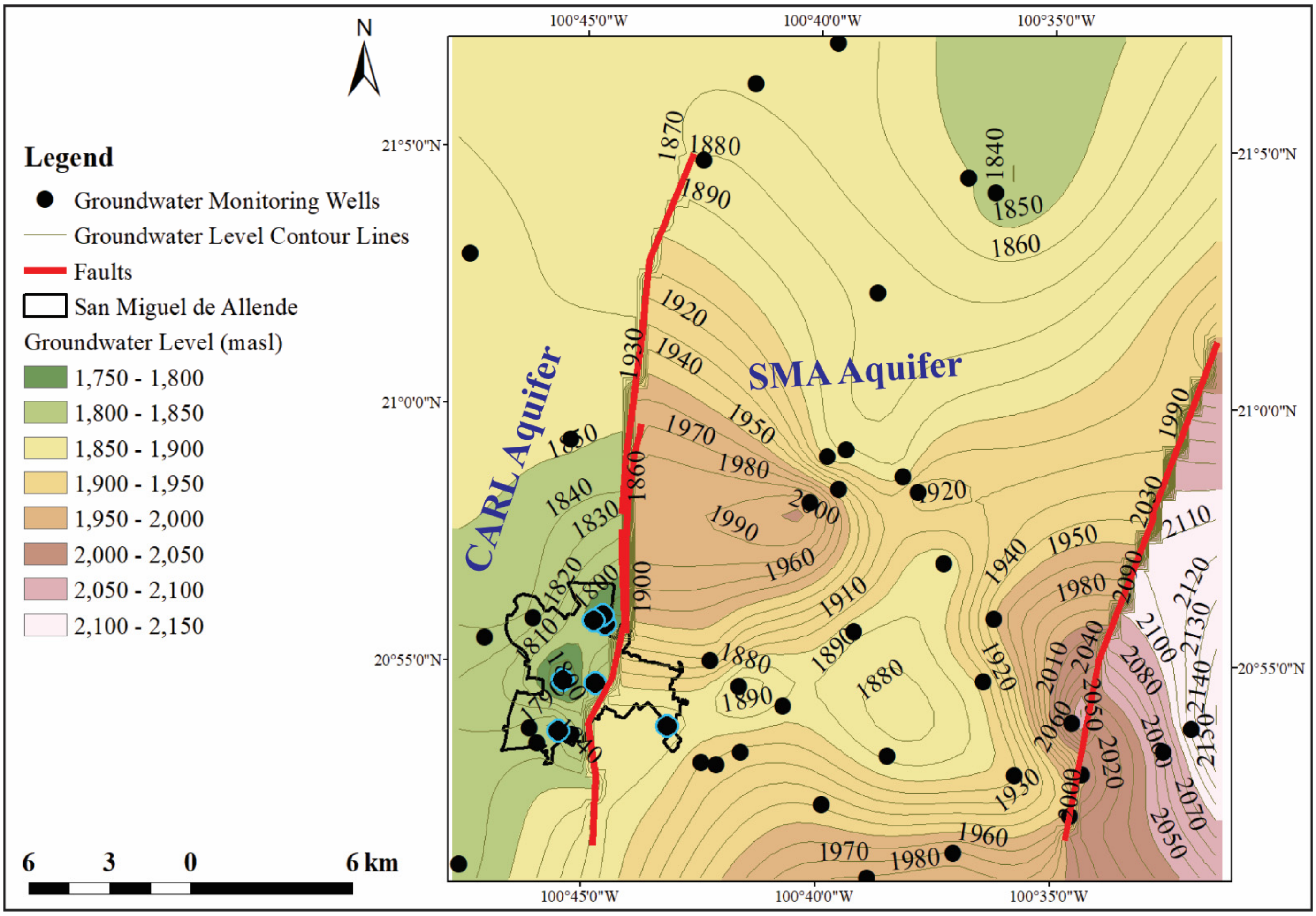

Figure S1. The distribution of groundwater level in CARL and SMA aquifers respectively. The black points with blue outline within the San Miguel de Allende (SMA) indicate seven selected time series wells. The observed groundwater level from these wells was obtained from the SMA's water municipality (SAPASMA). The observed groundwater level in other monitoring wells was obtained from the Guanajuato State Water Commission (CEGA, Comisión Estatal del Agua de Guanajuato) for the years 2008-2015.

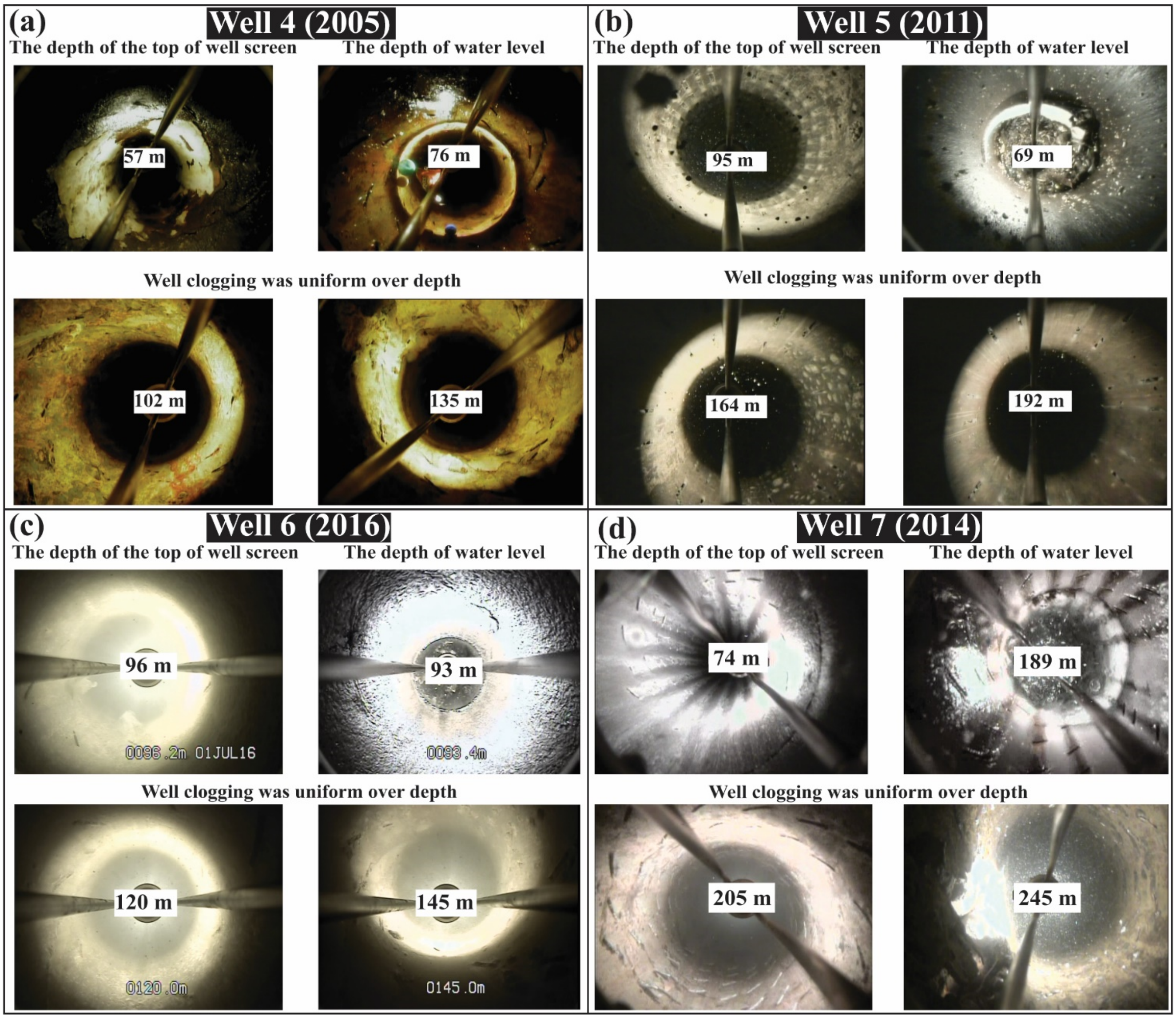

Figure S2. The snapshot of well log videos recorded from downhole camera. (a)-(d) represent Wells 4-7, respectively. Each panel includes four snapshots, which indicate the depth of the top of well screen, the depth of water level and well clogging, respectively. For the parathesis next to each well ID, it indicated the year that the well log video was recorded.

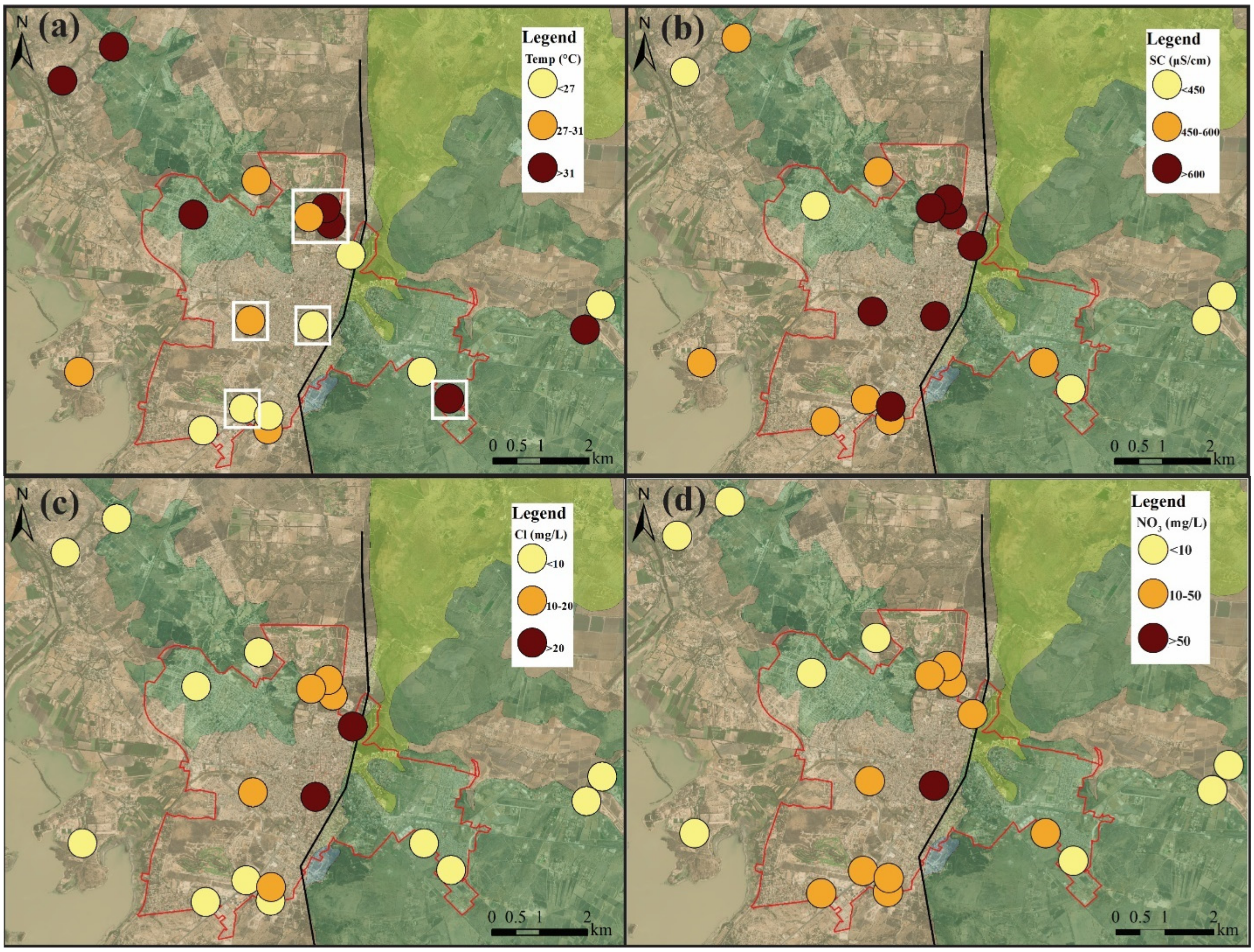

(a)
Legend
Temp (°C)
<27
27-31
>31
0 0.5 1 2 km
(b)
Legend
SC (µS/cm)
<450
450-600
>600
0 0.5 1 2 km
(c)
Legend
Cl (mg/L)
<10
10-20
>20
0 0.5 1 2 km
(d)
Legend
NO3 (mg/L)
<10
10-50
>50
0 0.5 1 2 km

continued

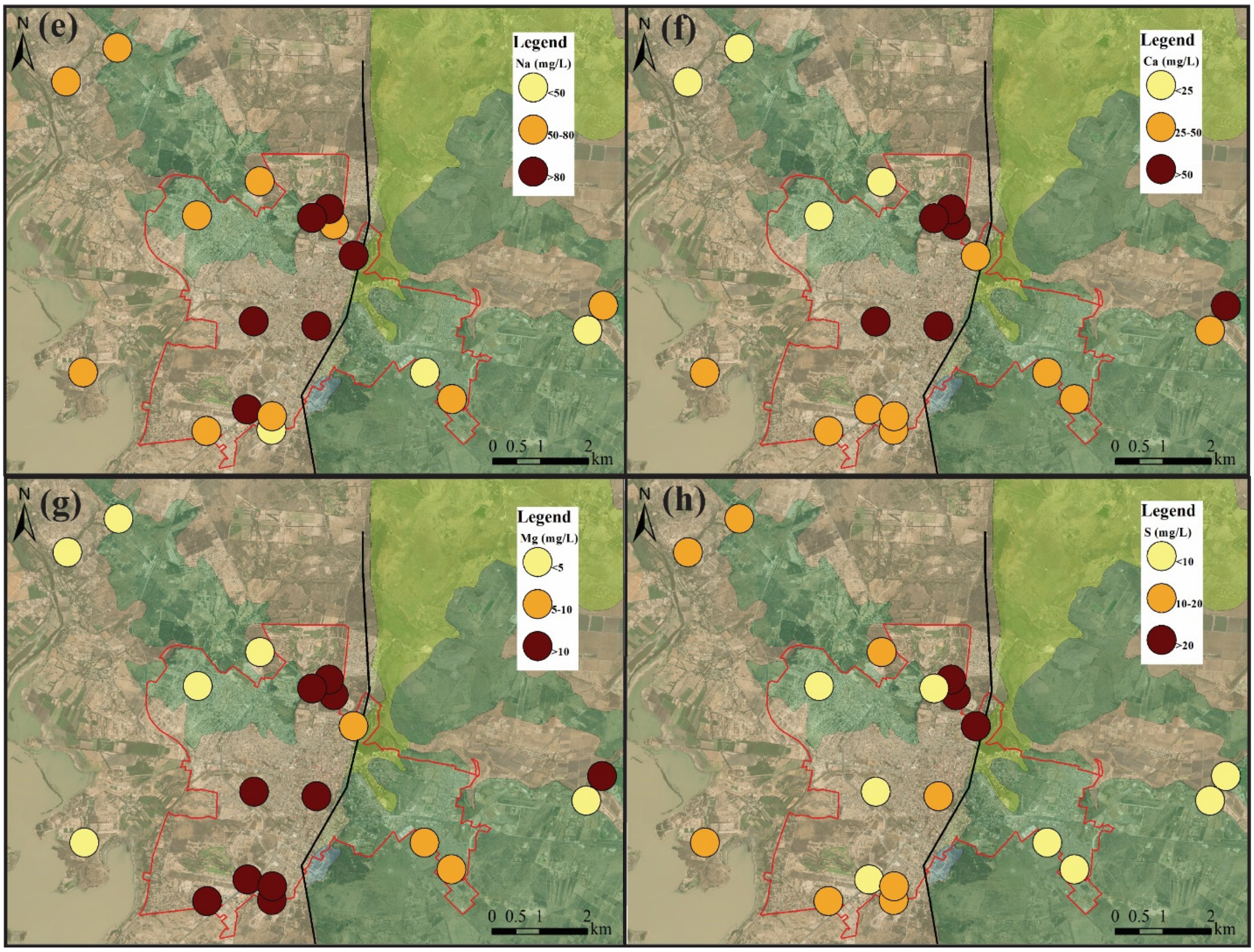
(e)
Legend
Na (mg/L)
<50
50-80
>80
0 0.5 1 2 km
(f)
Legend
Ca (mg/L)
<25
25-50
>50
0 0.5 1 2 km
(g)
Legend
Mg (mg/L)
<5
5-10
>10
0 0.5 1 2 km
(h)
Legend
S (mg/L)
<10
10-20
>20
0 0.5 1 2 km

continued

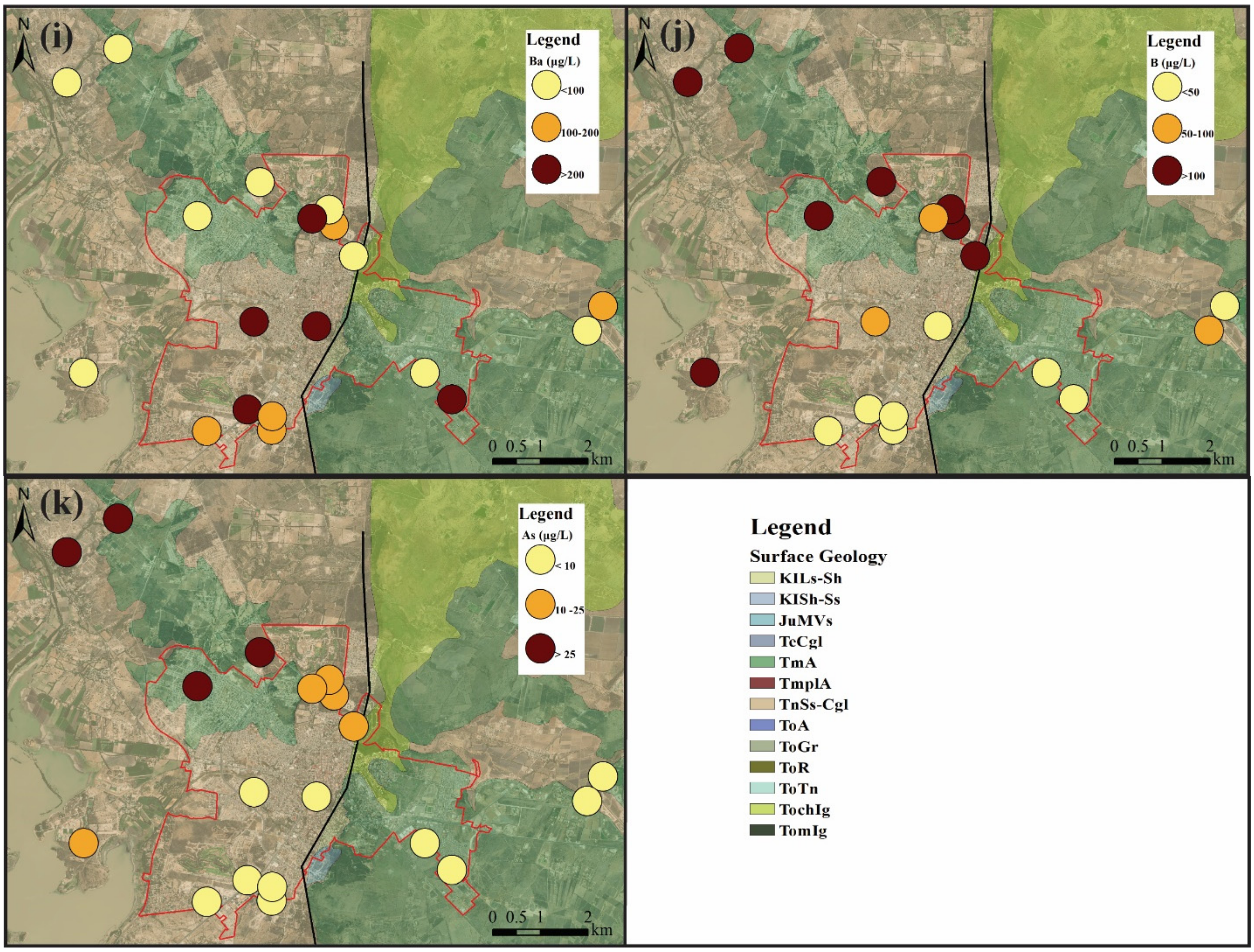

Figure S3. (a)-(k) represent the distribution of temperature (Temp), Specific Conductance (SC), chloride (Cl), nitrate (NO_3), sodium (Na), calcium (Ca), magnesium (Mg), sulfur (S), barium (Ba), boron (B) and arsenic (As) concentration in 19 wells across the SMA, respectively. In panel (a), the white squares highlight seven time series wells used to investigate the ambient flow Each panel is underlain with surface geology and land use maps. The surficial geology symbols mean: KILs-Sh: Lower Cretaceous Metamorphic Limestone; KISh-Ss: Lower Cretaceous Metamorphic shale interbedded with metamorphic sandstone; JuMVs: Lower Cretaceous Meta-volcanosedimentary; TeCgl: ertiary Eocene polymictic conglomerate; TmA: Tertiary Miocene Andesite; TmplA: Tertiary Miocene-Pliocene Andesite; TnSs-Cgl: Tertiary Neogene interbedded Sandstone and polymictic Conglomerate; ToA: Tertiary Oligocene Andesite; ToGr: Tertiary Oligocene Granodiorite; ToR: Tertiary Oligocene Rhyolite; ToTn: Tertiary Oligocene Tonalite; TochIg: Tertiary Oligocene Chattian Ignimbrite; TomIg: Tertiary Oligocene-Miocene Ignimbrite.

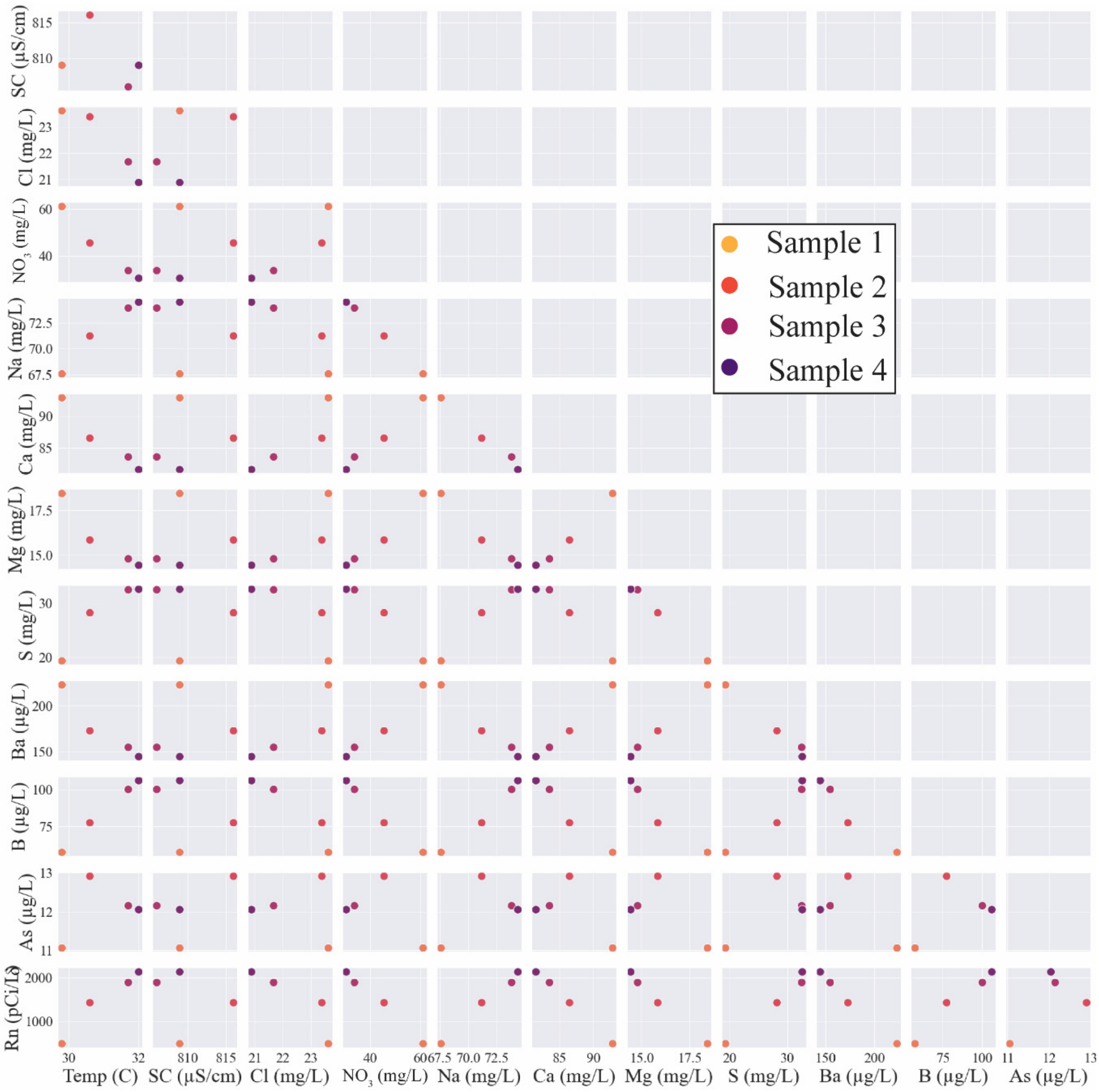

Figure S4. Correlation matrix of 12 tracers (Temp, SC, Cl, NO_3, Na, Ca, Mg, S, Ba, B, As and radon (Rn)) plotted against each other for Well 3 is presented to demonstrate the linear relationship between conservative tracers. Samples 1-4 indicate the water samples collected at the first wellbore volume (within 5 minutes), 30, 90 and 180 minutes after the start of pumping.

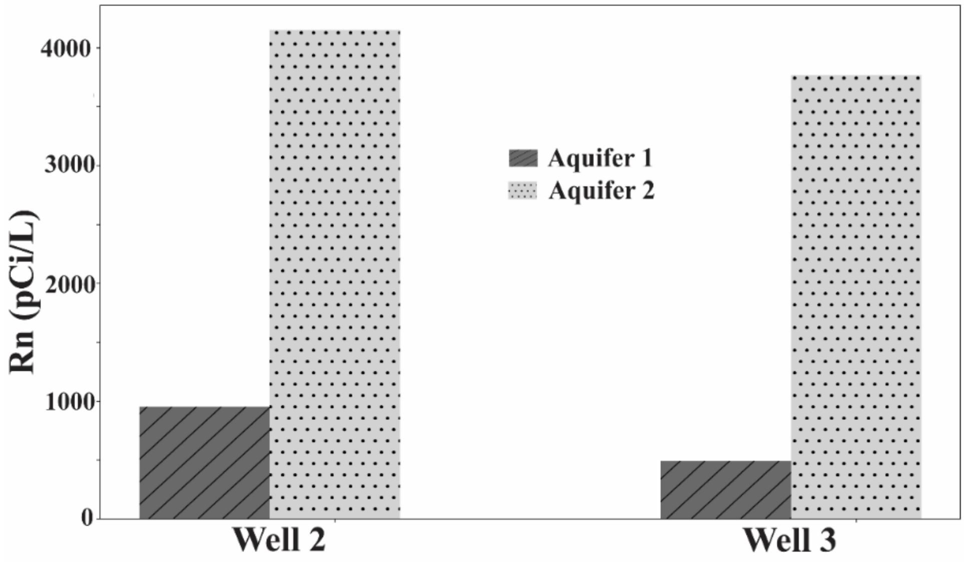

Figure S5. The radon (Rn) concentration in Aquifer 1 and Aquifer 2 at Wells 2 and 3.

Table S1. The concentration of conservative tracers in each well during a one-day's pumping. The values highlighted by light grey indicated the tracer's concentration didn't change in that well during the pumping. In this study, non-changing indicated the change of tracer's concentration in the well was less than 10% of corresponding changes in other wells.

Well ID	Sampling Schedule			Cl	NO_3	Na	Mg	Ca	S	As	B	Ba
	Time (min)	Total Volume (m^3)	# Wellbore Volume	mg/L						μg/L		
**1**	1	2	0.1	16.6	40.6	99.7	14.1	82.4	9.3	7.0	51.3	263.5
	40	84	7.0	16.8	41.1	99.7	14.2	82.9	9.3	6.9	49.9	258.1
	90	189	15.6	17.2	41.4	100.6	14.3	82.9	9.9	7.3	50.7	259.5
	195	410	33.9	16.7	40.9	101.4	14.6	83.0	9.5	7.2	49.1	261.2
	305	641	53.0	16.7	41.0	103.6	14.9	83.0	9.9	7.6	51.6	265.6
**2**	2	1	0.1	19.8	12.9	73.9	12.5	73.4	39.3	14.0	157.6	103.1
	15	20	2.5	20.4	13.1	82.0	12.1	74.0	45.0	16.4	171.0	88.3
	65	88	10.7	20.9	12.3	84.7	11.4	70.2	44.2	18.4	206.4	81.7
	245	331	40.2	20.9	12.3	85.0	11.3	69.3	42.9	17.5	210.0	83.0
**3**	1	1	0.1	23.7	61.3	68.5	18.4	92.9	15.9	9.0	46.2	223.1
	20	32	2.7	22.7	44.2	73.0	15.7	86.8	28.5	12.7	78.5	172.4
	110	178	15.1	21.6	33.1	74.5	14.7	83.5	32.7	12.5	97.3	154.6
	230	373	31.6	20.9	30.4	74.5	14.4	81.6	32.6	12.1	106.3	144.7
**4**	1	1	0.2	24.7	27.9	91.9	15.8	78.9	12.3	3.8	39.7	280.0
	40	70	8.4	15.8	26.1	96.1	11.6	60.3	9.6	5.1	63.4	277.9
	65	167	20.0	15.2	26.2	96.7	10.7	57.2	9.6	5.2	67.5	264.4
	170	299	35.8	15.0	26.1	97.2	11.4	58.4	9.7	5.4	70.1	264.5
	240	422	50.6	15.7	26.5	97.1	11.3	59.1	9.8	5.5	70.5	266.0
**5**	1	1	0.1	49.7	168.1	144.6	24.2	101.2	15.5	3.6	31.0	666.2
	46	64	5.1	48.3	163.7	146.0	23.9	100.0	15.3	3.6	29.4	657.3
	105	145	11.7	48.3	163.7	147.7	23.9	99.0	15.2	3.5	29.7	665.8
	205	283	22.7	48.1	163.8	151.0	24.0	99.6	15.4	3.6	29.0	667.8
	285	393	31.6	48.0	164.4	152.9	23.9	100.9	15.4	3.6	28.1	670.1
**6**	1	1	0.1	11.0	25.6	83.1	12.8	49.3	7.9	4.3	35.0	274.4
	45	54	5.8	10.2	25.1	83.6	12.4	48.3	7.9	4.2	34.0	270.3
	90	108	11.6	10.3	25.5	84.4	12.7	48.9	7.9	4.3	34.0	273.2
	180	216	23.2	10.1	25.3	85.6	12.5	49.1	7.9	4.2	33.5	277.3
	290	348	37.3	10.0	25.2	84.7	12.4	48.4	7.9	4.2	32.9	272.4
**7**	1	1	0.2	3.0	7.3	67.7	8.9	27.0	3.2	2.9	22.9	199.3
	40	39	11.6	3.0	7.2	66.9	8.7	26.1	3.1	2.4	18.8	196.3
	90	87	26.2	2.9	6.3	66.8	8.9	27.3	3.1	2.3	17.4	202.3
	165	159	48.0	2.9	6.5	66.6	8.9	27.4	3.2	2.4	17.6	202.0
	265	256	77.0	2.8	6.2	66.5	8.8	27.3	3.2	2.5	17.4	201.8

Table S2. The concentration of non-conservative tracers and water isotopes in each well during a one-day's pumping[1]. For each well, the sampling schedule is the same as the schedule presented in Table S1. Therefore, the sampling schedule is not included in this table. The values highlighted by light grey indicated its concentration didn't change during the pumping. In this study, non-changing indicated the change of tracer's concentration in the well was less than 10% of corresponding changes in other wells.

Well ID	F	SO_4	K	Si	P	Fe	$\delta^{18}O$	$\delta^{2}H$
	mg/L				μg/L		‰	
**1**	0.7	32.0	9.9	25.6	2.7	0.4	-8.9	-67.5
	0.7	32.5	11.7	25.2	2.5	0.5	-9.1	-68.8

	0.7	32.9	11.5	26.2	2.6	1.8	-9.2	-70.0
	0.7	32.3	11.7	25.2	2.5	0.5	-9.3	-70.5
	0.7	32.4	12.4	26.2	2.5	3.4	-9.2	-71.2
**2**	2.1	74.9	20.1	41.4	4.0	9.7	-9.5	-72.4
	2.1	83.9	20.1	40.1	3.6	6.1	-9.6	-72.2
	2.1	81.7	19.4	38.9	3.3	bdl[2]	-10.0	-77.5
	1.7	77.2	18.5	39.6	4.3	1.7	-10.1	-76.4
**3**	0.5	35.4	19.1	51.3	5.4	2.7	-9.4	-74.5
	1.0	52.0	21.4	48.3	1.1	bdl	-9.2	-71.5
	1.5	60.1	20.7	45.1	2.1	bdl	-9.4	-74.6
	1.5	59.7	20.4	44.0	5.2	bdl	-9.5	-70.9
**4**	0.4	42.5	12.7	30.8	18.9	bdl	-9.5	-72.1
	0.6	31.9	11.7	30.1	10.7	bdl	-9.4	-72.8
	0.6	31.6	3.1	28.2	9.7	bdl	-9.4	-72.7
	0.8	33.6	11.9	30.0	10.7	bdl	-9.3	-72.7
	0.6	31.9	11.6	29.8	11.1	bdl	-9.4	-72.6
**5**	0.2	52.6	10.6	31.5	10.3	73.4	-9.5	-73.3
	0.2	52.0	11.0	31.2	9.4	1.1	-9.5	-73.4
	0.2	52.0	11.6	31.3	9.1	1.3	-9.5	-73.8
	0.2	52.0	11.1	31.2	9.9	0.1	-9.7	-73.8
	0.3	52.7	11.1	31.2	10.0	bdl	-9.9	-74.1
**6**	0.3	27.1	10.8	34.9	8.0	0.5	-9.5	-71.9
	0.3	27.3	11.0	34.5	7.5	2.0	-9.4	-72.5
	0.3	27.7	11.3	35.0	7.6	0.5	-9.5	-72.8
	0.3	27.5	10.2	34.1	7.6	bdl	-9.5	-73.2
	0.3	27.4	11.3	34.2	8.7	bdl	-9.3	-73.6
**7**	0.2	10.3	10.0	28.9	3.5	10.2	-11.5	-81.0
	0.2	10.3	9.8	28.8	3.9	11.9	-10.7	-77.5
	0.2	10.2	10.1	29.0	3.6	8.0	-10.4	-77.1
	0.2	10.2	9.9	29.1	3.4	6.1	-10.2	-76.3
	0.2	10.3	10.1	28.8	4.3	4.3	-10.3	-75.4

[1]: The measured concentration of Li and Br were below detection limit. Therefore, these two elements were not included in the table.

[2]: bdl: below detection limit.

Table S3. The radon (Rn) concentrations measured in Wells 2 and 3. The radon concentration was not measured in other wells. For Wells 2 and 3, the sampling schedule is the same as the schedule presented in Table S1.

Well ID	Concentration (pCi/L)
**2**	952
	1694
	1608
	2553
**3**	491
	1428
	1889
	2130

Table S4. The root mean square error (RMSE) between observed and fitted concentrations of conservative tracers in 7 wells.

Well ID	Temperature (°C)	Specific Conductance (μS/cm)	Cl	NO_3	Na	Mg	Ca	S	As	B	Ba
			mg/L					μg/L			
**1**	0.0	1.6	0.1	0.1	0.1	0.0	0.0	0.2	0.1	0.4	0.5
**2**	0.1	29.1	0.0	0.1	0.0	0.0	0.5	0.1	0.0	0.4	0.0
**3**	0.1	2.0	0.0	0.0	0.0	0.0	0.0	0.0	0.0	0.0	0.0
**4**	0.1	2.1	0.2	0.1	0.1	0.2	0.1	0.0	0.0	0.4	2.2
**5**	0.0	6.4	0.0	0.1	0.0	0.0	0.1	0.1	0.0	0.2	1.9
**6**	0.0	3.8	0.1	0.1	0.1	0.1	0.3	0.0	0.0	0.1	2.2
**7**	0.0	1.5	0.0	0.2	0.0	0.1	0.4	0.0	0.0	0.1	1.6

Table S5. The coefficient of determination (R^2) between observed and fitted concentrations of conservative tracers in 7 wells.

Well ID	Temperature (°C)	Specific Conductance (μS/cm)	Cl	NO_3	Na	Mg	Ca	S	As	B	Ba
			mg/L					μg/L			
**1**	0.61	0.96	0.50	0.80	0.99	1.00	0.99	0.57	0.81	0.79	0.97
**2**	0.97	0.81	1.00	0.91	1.00	1.00	0.94	1.00	1.00	1.00	1.00
**3**	0.98	0.92	1.00	1.00	1.00	1.00	1.00	1.00	1.00	1.00	1.00
**4**	0.97	0.99	1.00	0.98	1.00	0.99	1.00	1.00	1.00	1.00	0.90
**5**	0.82	0.74	1.00	1.00	1.00	0.87	0.99	0.66	0.92	0.93	0.81
**6**	0.94	0.84	0.98	0.54	1.00	0.68	0.43	0.04	0.67	0.98	0.13
**7**	0.95	0.88	0.85	0.82	1.00	0.09	0.38	0.96	1.00	1.00	0.50

Table S6. The estimated temperature (Temp) and concentration of conservative constituents in Aquifer 2 when the ratio of B_1/B_2 equaled to 0.7 and 1.2, respectively[1].

Well ID	Temp	SC[2]	Cl	NO3	Na	Ca	Mg	S	Ba	B	As	Rn
	°C	μS/cm	mg/L						μg/L			pCi/L
**1**	33.7	788.2	19.4	13.4	78.3	75.3	12.2	39.9	101.2	133.4	13.3	
	33.3	791.5	19.6	16.5	77.6	76.4	12.6	38.6	109.1	128.5	13.2	
**2**	35.7	519.8	21.5	8.9	89.2	61.5	9.5	43.7	63.3	329.3	23.1	4838.9
	35.3	536.5	21.4	9.1	88.3	62.2	9.7	43.4	65.8	317.9	22.5	3886.3
**3**	30.2	650.8	16.8	41.6	108.6	83.8	15.9	10.7	268.2	52.0	8.4	4471.4
	30.2	661.2	16.8	41.4	106.9	83.5	15.6	10.4	267.3	51.9	8.2	3495.8
**4**	29.9	534.3	2.8	24.7	103.9	32.7	5.8	6.5	248.5	108.2	7.4	
	29.1	585.5	6.9	25.3	101.7	41.3	7.7	7.5	254.3	95.5	6.7	
**5**	32.2	412.7	2.7	5.5	65.1	28.0	9.1	3.4	205.4	11.0	1.7	
	32.0	416.7	2.8	5.8	65.6	27.8	9.1	3.4	204.2	13.2	1.9	
**6**	27.4	559.5	8.9	24.9	86.7	47.2	12.2	7.9	269.9	35.7	4.2	
	26.4	575.3	9.3	25.0	86.1	47.6	12.3	7.9	270.8	35.0	4.2	
**7**	23.5	1114.0	47.3	158.3	159.0	97.7	23.5	15.4	669.7	26.4	3.5	
	23.5	1125.7	47.8	160.1	156.3	98.3	23.7	15.4	669.1	27.2	3.6	

[1]: for each well, the first and second row indicated the estimated concentration when the ratio of equals to 0.7 and 1.2, respectively.

[2]: SC indicated specific conductance.

Table S7. The estimated volume (m^3) of ambient flow from each conservative tracer. Hot and cool colors indicate high and low ambient flow volumes, respectively.

Well ID	Temp[1]	SC[2]	Cl	NO_3	Na	Mg	Ca	S	As	B	Ba	Rn
1	14.4	112.1	50.1	**170.6**	278.0	173.6	39.7	121.4	296.6	354.7	320.2	-
2	59.5	3.8	7.0	342.4	17.1	151.7	342.9	**65.8**	257.7	**64.5**	32.6	346.8
3	**63.9**	54.7	116.7	54.5	11.0	47.6	86.6	7.5	105.2	86.4	**67.3**	86.6
4	45.9	30.7	1.9	37.9	**31.8**	12.1	5.9	8.7	39.7	36.3	176.9	-
5	117.3	82.8	26.5	11.7	87.7	24.9	120.5	55.3	28.9	**60.3**	259.2	
6	47.9	27.7	25.9	5.6	102.4	**46.7**	3.0	5.5	154.2	66.1	245.7	
7	16.0	26.9	32.2	32.6	**27.8**	134.5	44.2	234.8	16.3	12.4	19.6	

For each well, the bold value and underline value indicates the median value and the one falls within the interquartile range of ambient flow volumes estimated from conservative tracers. Rn concentration was only measured in Wells 2 and 3.

[1]: temp indicated temperature.

[2]: SC indicated specific conductance.

Table S8. The average daily pumping hours for each month in 2018.

Well ID	Jan.	Feb.	Mar.	Apr.	May	Jun.	Jul.	Aug.	Sep.	Oct.	Nov.	Dec.
1	17.9	19.1	17.9	18.8	11.4	21.1	21.1	21.0	17.1	20.7	18.9	19.0
2	14.6	13.9	17.2	15.4	15.7	14.4	15.7	13.5	13.7	13.7	14.6	14.4
3	13.4	13.1	15.7	11.4	12.2	8.1	7.2	6.6	5.0	6.1	10.9	11.7
4	8.5	8.9	8.6	8.5	9.3	9.3	9.0	8.7	9.2	9.4	9.7	9.2
5	6.5	10.7	12.3	14.1	12.3	12.1	10.5	12.6	3.9	3.8	3.8	9.8
6	10.9	5.0	4.7	5.5	15.3	14.7	14.1	12.6	14.3	10.3	5.3	5.5
7	19.5	16.5	9.3	9.9	10.0	14.2	20.5	20.8	16.6	17.1	15.9	8.0

Text S1. Pumping Test Analysis and Estimated Aquifer Parameters

S1.1. Pumping Test Analysis

The obtained drawdown curve in the observation well was analyzed using both numerical and analytical models to solve for aquifer transmissivity (T) and storativity (S). The 3-D numerical groundwater model was developed using the code for MODFLOW 2000 within the software Groundwater Modeling System (GMS 10.6, Aquaveo, LLC). The long-screened well was represented by the multi-node well package (MNW2) within GMS. The total thickness of the aquifer in the model was set at 400 m which more than covers the extent of the pumped interval of the aquifer. Knappett et al. (2018) [1] suggested that the SMA fault prevents the lateral mixing of groundwaters. In the model, therefore, the fault, which is located 800 m east of Wells 2 and 3, is treated as a no-flow boundary in the model. Other boundaries (north, south and west boundaries) were designated as constant head boundaries. The observed water levels in Well 1 were imported into the model. The first observation was used as the starting head of the domain. The built-in parameter estimation package (PEST) was used to estimate aquifer parameters by inverse modeling.

The analytical model was based on the Theis well function [2] which describes transient drawdown in hydraulic heads owing to pumping. This model was fit to the observed drawdown in Well 3. The method of images was used to mimic the no-flow boundary at the fault by placing two imaginary pumping wells on the opposite side and equidistant from the fault as Wells 2 and 3. The image wells had the same pumping rates as Wells 2 and 3, respectively. The optimal values of T and S were determined by manually minimizing the root mean square error (RMSE) between observed and modeled drawdown.

S1.2. Estimated T and S from Pumping Test

The hydraulic properties of the CARL aquifer in the vicinity of three of the study wells were needed to provide more context for the mixing model. The optimal values of T and S were determined with the analytical model by choosing the minimum of root mean square error (RMSE) across reasonable parameter space. The minimum RMSE (0.003 m) was found at $T = 2.8 \times 10^4$ m^2/day and $S = 1.8 \times 10^{-4}$ (Fig. S6a). The uniqueness of this solution for T and S was estimated by extracting the oval shape that results from the intersection of the RMSE response surface and the horizontal plane of RMSE = 0.01 m. This threshold was chosen because it is the approximate level of accuracy of one physical measurement of the water table. Using this technique, the ranges of T and S are 2.1-3.8 × 10^4 m^2/day and 0.7-4.0 × 10^{-4}, respectively.

For comparison, the optimal values of *T* and *S* determined from the automated PEST procedure implemented within the numerical model were 1.7 × 10^4 m^2/day and 1.8 × 10^{-4}, respectively, which had the RMSE of 0.006 m (Fig. S6c). Predicted drawdowns by both models are presented against that observed (Fig. S6c). The numerical model simulated drawdown best during the early and late stages of the pumping test. In contrast, the analytical model simulated drawdown best during the middle stage. Furthermore, the numerical model more accurately described the timing when the drawdown started decline than the analytical one.

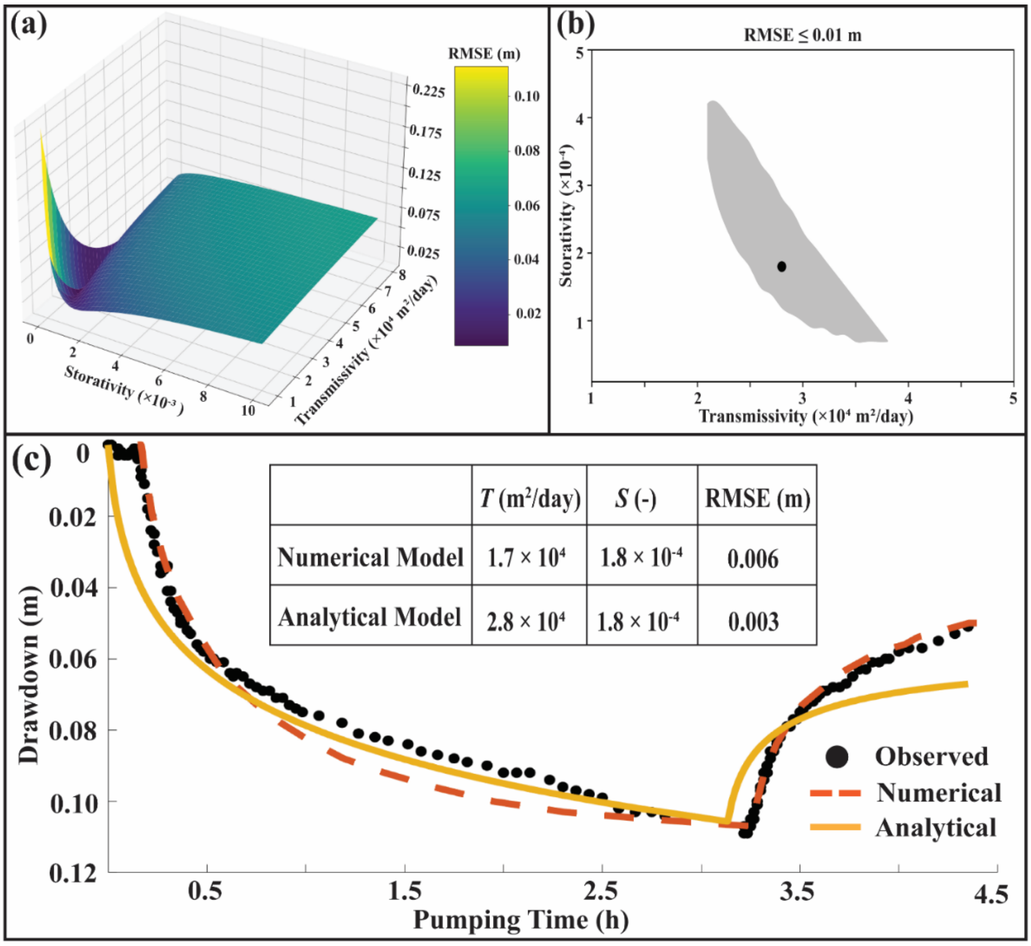

Figure S6. Fitting results of the pumping test observations by the analytical and numerical models. (a) the root mean square error (RMSE) response surface generated by the analytical model across the 2-D space created by varying transmissivity (*T*) and storativity (*S*) across reasonable values. (b) the range of *T* and *S* across which the analytical model fit has an RMSE of is less than or equal to 0.01 m. The red point indicates the optimal estimate of *T* and *S*. (c) the observed drawdown and modeled drawdown from numerical and analytical models, respectively. The best fitted values of *T* and *S* from each model are presented in the table.

S1.3. Discussion

Although the total thickness of local aquifer is unknown, Mahlknecht et al. (2006) [3] estimated that it was up to 400-500 m in some places. There is substantial geologic heterogeneity across the city but for the present we will assume the saturated thickness of the aquifer is about 300 m which includes the part of the aquifer that is being accessed by all study wells (Table 1). Based on the estimated *T* of the sedimentary portion of the CARL aquifer, the value of *K* derived from numerical and analytical models was 57 and 93 m/day, respectively. In SMA, even the entire Independence Basin, aquifer properties (*K* and *S*) are rarely reported in previous publications. Moreover, those reported values presented great variations (0.2 -10 m/day from CONAGUA (2020) [4] and 5 – 430 m/day from Mahlknecht et al. (2006) [5]). Hydraulic conductivities were also reported for similar lithologies in the region. In the city of Querétaro, which is located approximately 50 km southeast of SMA, the local aquifer system is heavily faulted and consists of volcanic rocks and sedimentary material [6]. Carrera-Hernández et al. (2016) [6] used 0.01-2 m/day as the range of local *K* value but didn't give any information about the source of this range. In the aquifer with widespread faults

and fractures, the value of K is related to fracture characteristics, such as fracture aperture and frequency, fracture length and fracture interconnectivity [7-9]. Therefore, in this region K ranges over approximately 2 orders of magnitude even across similar types of geologic material and faulting.

In terms of the storativity of the aquifer in SMA, to the author's knowledge, a detailed measurement of S has not been reported in any publications. Values of S in surrounding areas have been reported, however. Based on the consulting work conducted in 1981, the S of the aquifer in the upper basin of the Laja River was estimated as 0.05-0.06 [4]. Carrera-Hernández et al. (2016) [6] reported 6.9×10^{-5} (m^{-1}) as the specific storage of aquifer system in the city of Querétaro, which has similar lithology as the aquifer in SMA. This reported value is equivalent to the S of 0.02 if the saturated thickness of the aquifer is assumed to be 300 m. These values are 100 times higher than the value of S derived from this study. Unfortunately, detailed information about the method produced those reported values was not provided. Therefore, more work needs to be conducted to estimate the value of S in the SMA area more accurately. It is important to note that the aquifers throughout this region are known to be unconfined. The low S values measured in the pumping test, however, suggests a confined aquifer. This apparent controversy can be resolved by considering the short duration of the pumping test and the large scale of the pumping test. Internal fine layers within sedimentary rock or sediments increase hydraulic anisotropy of the aquifer as does the scale of measurement. This causes pressure changes to propagate through an aquifer rapidly, as though the aquifer diffusivity (T/S) was high [10]. So even within a single physical aquifer, sufficient internal anisotropy will create differences in the drawdown response in early time (<3 hours) and late time (>1 day). In this case, there was not enough storage space for the produced water to run the pumping wells for more than several hours so we were limited to a short duration pumping test which may have biased the measurement of S [11].

References:

1. Knappett, P. S., Li, Y., Hernandez, H., Rodriguez, R., Aviles, M., Deng, C., Piña, V., Giardino, J. R., Mahlknecht, J., Datta, S. 2018. Changing recharge pathways within an intensively pumped aquifer with high fluoride concentrations in central mexico. Science of the Total Environment, 622, 1029-1045.
2. Theis, C. V. 1935. The relation between the lowering of the piezometric surface and the rate and duration of discharge of a well using ground-water storage. Eos, Transactions American Geophysical Union, 16, 519-524.
3. Mahlknecht, J., Gárfias-Solis, J., Aravena, R., Tesch, R. 2006. Geochemical and isotopic investigations on groundwater residence time and flow in the independence basin, mexico. Journal of Hydrology, 324, 283-300.
4. Conagua 2020. Actualización de la disponibilidad media anual de agua en el acuífero san miguel de allende (1107) estado de guanajuato. *Comision Nacional del Agua.*
5. Mahlknecht, J., Medina-Mejía, M. G., Gárfias-Solis, J., Cano-Aguilera, I. 2006. Intrinsic aquifer vulnerability assessment: Validation by environmental tracers in san miguel de allende, mexico. Environmental Geology, 51, 477-491.
6. Carrera-Hernández, J., Carreón-Freyre, D., Cerca-Martínez, M., Levresse, G. 2016. Groundwater flow in a transboundary fault-dominated aquifer and the importance of regional modeling: The case of the city of querétaro, mexico. Hydrogeology Journal, 24, 373-393.
7. Hamm, S.-Y., Kim, M., Cheong, J.-Y., Kim, J.-Y., Son, M., Kim, T.-W. 2007. Relationship between hydraulic conductivity and fracture properties estimated from packer tests and borehole data in a fractured granite. Engineering Geology, 92, 73-87.
8. Tan, J., Rong, G., Zhan, H., He, R., Sha, S., Li, B. 2020. An innovative method to evaluate hydraulic conductivity of a single rock fracture based on geometric characteristics. Rock Mechanics and Rock Engineering, 53, 4767-4786.
9. Zimmerman, R. W., Bodvarsson, G. S. 1996. Hydraulic conductivity of rock fractures. Transport in Porous Media, 23, 1-30.
10. Shuai, P., Knappett, P. S. K., Hossain, S., Hosain, A., Rhodes, K., Ahmed, K. M., Cardenas, M. B. 2017. The impact of the degree of aquifer confinement and anisotropy on tidal pulse propagation. Groundwater, 55, 519-531.
11. Neuman, S. P. 1972. Theory of flow in unconfined aquifers considering delayed response of the water table. Water Resources Research, 8, 1031-1045.
